# Alismatis Rhizoma methanolic extract—Effects on metabolic syndrome and mechanisms of triterpenoids using a metabolomic and lipidomic approach

**DOI:** 10.3389/fphar.2022.983428

**Published:** 2022-09-09

**Authors:** Li Jia, Min Zhang, Pengli Wang, Liming Wang, Peng Lei, Ruijiao Du, Lifeng Han, Peng Zhang, Yuefei Wang, Miaomiao Jiang

**Affiliations:** ^1^ State Key Laboratory of Component-based Chinese Medicine, Tianjin University of Traditional Chinese Medicine, Tianjin, China; ^2^ Haihe Laboratory of Modern Chinese Medicine, Tianjin, China

**Keywords:** metabolic syndrome, Alismatis rhizoma extract, fructose, metabolomics, lipidomics

## Abstract

Alismatis rhizoma is a traditional Chinese medicine. Studies have demonstrated that Alismatis rhizoma also has therapeutic effects on metabolic syndrome. However, the pharmacodynamic material basis and mechanism are still unclear. First, UHPLC/Q-Orbitrap MS was used to detect the chemical components of the Alismatis rhizoma extract, and 31 triterpenoids and 2 sesquiterpenes were preliminarily identified. Then, to investigate the mechanism of the Alismatis rhizoma extract on metabolic syndrome, a mouse model of metabolic syndrome induced by high-fructose drinks was established. The results of serum biochemical analysis showed that the levels of TG, TC, LDL-C, and UA after the Alismatis rhizoma extract treatment were markedly decreased. ^1^H-NMR was used to conduct non-targeted metabolomics studies. A total of 20 differential metabolites were associated with high-fructose–induced metabolic syndrome, which were mainly correlated with 11 metabolic pathways. Moreover, UHPLC/Q-Orbitrap MS lipidomics analysis found that a total of 53 differential lipids were screened out. The results showed that Alismatis rhizoma extract mainly reduces the synthesis of glycerophospholipid and ceramide and improves the secretion of bile acid. This study shows that the Alismatis rhizoma extract can treat metabolic syndrome mainly by inhibiting energy metabolism, amino acid metabolism, and regulating bile acid to reduce phospholipid content.

## 1 Introduction

Metabolic syndrome refers to a pathophysiological state in which obesity, dyslipidemia, insulin resistance, hypertension, hyperuricemia, and other metabolic risk factors occur simultaneously (Eva et al., 2011; Hoffman et al., 2014; Samson and Garber, 2014; Targher and Byrne, 2015; Rochlani et al., 2017). More precisely, metabolic syndrome can be diagnosed when there are three of the following five risk factors: central obesity, hypertension, hyperglycemia, high triglyceride, and low- and high-density lipoproteins (Kanwar and Kowdley, 2016). Metabolic syndrome may lead to nonalcoholic fatty liver disease (NAFLD), type 2 diabetes mellitus (T2DM), cardiovascular disease (CVD), chronic kidney disease, gout, and other such diseases (Velasquez, 2018). Since fructose bypasses the rate-limiting step catalyzed by phosphofructokinase in the liver, resulting in a high burden of *de novo* lipogenesis and uric acid production, a large intake of fructose can induce metabolic syndrome. Recent studies have shown that excessive intake of fructose is the main cause of metabolic syndrome, which may be related to fat accumulation, oxidative stress, and inflammation caused by fructose (Miller et al., 2008; Shao and Khew-Voon, 2011; Lakhan and Kirchgessner, 2013). At present, the prevalence of metabolic syndrome is gradually increasing all over the world. Although some drugs targeting metabolic syndrome or its components are currently available, they have prominent side effects (Jalal et al., 2010; Wu et al., 2020a). There is an urgent demand for safe and effective new antimetabolic syndrome drugs.

Alismatis rhizoma (Ze Xie in Chinese, Takusha in Japanese, and Taeksa in Korean) (Jang and Lee, 2021), derived from the dried tubers of *Alisma plantago-aquatica* L*.* of the Alismataceae family, has been recorded by Zhongjing Zhang of the Eastern Han dynasty in “Jin Gui Yao Lue” (Synopsis of Prescriptions of the Golden Chamber) (Song, 2009) and Li Shizhen of the Ming Dynasty in “Compendium of Materia Medica” (Li and Luo, 2003). Its traditional efficacy mainly includes diuretic and hypolipidemic therapy. It has been used for treating wat swelling, phlegm retention vertigo, and hyperlipidemia in clinical settings (Jeong et al., 2016). Previous research has suggested that Alismatis rhizoma extract can effectively regulate liver lipid (Tian et al., 2014) and sugar metabolism (Li and Qu, 2012), control liver injury (Zhang et al., 2017a), and have anti-inflammatory (Xue et al., 2014), anti-oxidative stress (Min-Kyung et al., 2015), anti-fibrosis (Shu et al., 2016), and other effects (Jeong et al., 2016). These indicate that Alismatis rhizoma also has therapeutic effects on metabolic syndrome. But the mechanism of the Alismatis rhizoma extract in treating metabolic syndrome induced by high-fructose drinks remains unclear.

In this study, high-fructose drink was used to induce metabolic syndrome in C57BL/6J mice, and Alismatis rhizoma extract was used for intervention. The chemical components are characterized by UHPLC/Q-Orbitrap-MS (Thermo Fisher Scientific, United States), thus providing a material basis for further pharmacodynamic analysis. The protective effect of the Alismatis rhizoma extract on high-fructose–induced liver injury was evaluated from serum, liver biochemical indicators, and liver pathological sections. The potential biomarkers of high-fructose–induced liver injury were screened based on ^1^H-NMR liver metabonomics, lipidomics of UHPLC/Q-Orbitrap-MS combined with multivariate statistical analysis, and to explore the possible mechanism of the Alismatis rhizoma extract in the treatment of metabolic syndrome.

## 2 Materials and methods

### 2.1 Plant material and extraction

Alismatis rhizome was purchased from Beijing Tong Ren Tang Pharmaceutical Co., Ltd (Fujian, China, No. 20180808) and identified as the dried stem tubers of *Alisma plantago-aquatica* L*.* by Dr. Honghua Wu, Associate Researcher (Tianjin University of Tradition Chinese Medicine). The plant's name has been checked with http://www.theplantlist.org. on March 7, 2022. The voucher specimen was deposited in the authors’ laboratory at the Tianjin University of Traditional Chinese Medicine (Tianjin, China, No. TJUTCM-AO-20180808). The extraction process is shown in Supplementary Figure S1. Alismatis rhizoma (7 kg) was immersed in 70 L of methanol for 3 days, and filtered and concentrated under reduced pressure, and the residues were extracted twice under heating and reflux with of 70% methanol, which was 10 folds the weight of the residues, and filtered and concentrated under reduced pressure, and the extracted concentrated solutions were combined and lyophilized to obtain the Alismatis rhizoma extract.

### 2.2 Reagents and materials

The reference compounds (alisol C 23-acetate, alisol B 23-acetate, alisol F, alisol F 24-acetate, and alismalactone 23-acetate) were prepared by laboratory separation. Pioglitazone was purchased from Shanghai Yuanye Bio-Technology Co., Ltd (Shanghai, China). Fructose and sodium carboxymethylcellulose were purchased from Dalian Meilun Biotechnology Co., Ltd (Dalian, China). Formalin, D_2_O, and 2,2,3,3-*d*_4_-3-(trimethylsilyl) propionic acid sodium salt (TSP-*d*_4_) were purchased from Cambridge Isotope Laboratories (Cambridge, FL, United States). Acetonitrile, methanol, and formic acid (Fisher, Fair Lawn, NJ, United States) were of UHPLC grade. Ultrapure water (18.2 MΩ• m at 25°C) was prepared in-house using a Milli-Q Integral 5 water purification system (Millipore, Bedford, MA, United States).

### 2.3 Preparation of extract and UHPLC/Q-Orbitrap-MS analysis

Accurately weighed 2.00 mg freeze-dried powder of the Alismatis rhizoma extract was dissolved in 1 ml methanol and then dissolved ultrasonically for 10 min, followed by vortexing for 5 min. The solution was centrifuged at 13,200 × *g* for 10 min, and the supernatant was taken as the test solution for the Alismatis rhizoma extract sample. An amount of 1.00 mg of the accurately weighed powder of eight reference standards was dissolved in 1 ml methanol and then mixed with the eight reference standard solutions and diluted to obtain a mixed standard sample, wherein the concentration of each standard was 100 μg/ml, and the supernatant was taken as the mixed standard sample.

The chemical components were analyzed using an UltiMate® 3000 UHPLC system coupled with a Q Exactive™ Q-Orbitrap™ Mass Spectrometer (Thermo Fisher Scientific, San Jose, CA, United States). Chromatographic separation was achieved on a BEH C18 column (2.1 × 50 mm, 1.7 µm; Waters) maintained at 30°C using a binary mobile phase which consisted of 0.1% FA in water (A) and acetonitrile (B). The mobile phase flow rate was set at 0.2 ml/min according to an optimal gradient eluting program: 0–3 min 40%–50% (B), 3–15 min 50–70% (B), and 15–19 min 70–95% (B). An injection volume of 2 µl was set. High-resolution MS data were recorded on the Q Exactive™ Quadrupole-Orbitrap™ Mass Spectrometer both in the positive and negative modes. The source parameters were spray voltage, −3.0 kV/+3.5 kV; capillary temperature, 350°C; gas temperature, 350°C; sheath gas flow rate, 35 arb; auxiliary gas flow rate, 10 arb; and sweep gas flow rate, 0 arb. The Orbitrap analyzer scanned over *m/z* 200–1500 normalized collision energy (NCE) at 10 V, 30 V, and 50 V. Data acquisition and processing were controlled and performed by the Xcalibur™ 4.1 software.

### 2.4 Animals and sample collection

Male C57BL/6J mice (aged 6–8 weeks) were obtained from Beijing Vital River Laboratory Animal Technology Co., Ltd (Beijing, China). All the mice were reared in the same breeding room at 23 ± 2°C and 50 ± 10% humidity with alternating 12-h light/dark cycles. After adaptive breeding for 7 days, 60 mice were divided into 5 groups (12 mice per group), which included the control group (C, equivalent physiological saline), model group (M, equivalent physiological saline), positive group (pioglitazone, PL, 6.00 mg/kg), Alismatis rhizoma extract low-dose group (LAO, 0.75 g/kg/d), and Alismatis rhizoma extract high-dose group (HAO, 1.50 g/kg/d) group. The animal dose was calculated according to the clinical dose given in the Chinese Pharmacopoeia. All groups were given ordinary feed. For the first 4 weeks, the mice in the control group drank freely, while mice in the model and administration groups were given a 15% high-fructose drink. The body weight of mice was monitored once a week. After 9 weeks, the mice were then anesthetized with a dose of 0.3%, 0.1 ml/10 g pentobarbital after 12 h of fasting, and the samples were collected. The blood samples were collected to prepare the serum for biochemical analysis. The livers were carefully dissected out, and the fat and connective tissues removed. After washing with cold PBS, the liver samples were immediately frozen in liquid nitrogen followed by storage at −80°C until further processing and analysis. All protocols used in this study followed the National Institutes of Health Guide for the Care and Use of Laboratory Animals and were approved by the Animal Ethics Committee of Tianjin University of Traditional Chinese Medicine (Ethics approval number TCM-LAEC2020019).

### 2.5 Histopathological and biochemical analysis

The liver tissue blocks of approximately 1 cm^3^ in size were placed in 10% formalin solution, embedded in paraffin wax, and cut into sections for hematoxylin and eosin (H&E) staining. Serum triglyceride (TG), serum cholesterol (TC), serum high-density lipoprotein (HDL-C), serum low-density lipoprotein (LDL-C), and serum uric acid (UA) were detected using an automatic biochemical analyzer; the liver biochemical indexes which included lipopolysaccharide (LPS) and tumor necrosis factor (TNF-α) were also analyzed by ELISA kits.

### 2.6 Metabolomics analysis of hepatic tissue based on ^1^H-NMR

The liver tissue (100 mg) was extracted with 400 μl of precooled methanol/water (*v/v* = 2:1) and was fully homogenized by a rapid tissue cell disrupter (FLUKO-F8, Shanghai, China) with dry ice and then subjected to centrifugation (13,200 × *g*, 4°C, 10 min). The resultant supernatant was dried under nitrogen and reconstituted into 550 μl of 0.15 M phosphate buffer in D_2_O (K_2_HPO_4_/NaH_2_PO_4_ = 4:1, pH = 7.45) containing 0.001% TSP-*d*_4_ (Wang et al., 2022). After centrifugation, 500 μl of the supernatant was transferred into a 5-mm NMR tube for analysis.

The ^1^H-NMR spectra for metabolites were acquired by using a CPMGPR1D pulse sequence at 303 K on a Bruker AVIII NMR 600 MHz spectrometer (proton frequency at 600.13 MHz) equipped with an automatic sampling system. A total of 64 scans were accumulated at a spectral width of 9615.4 Hz, and the 90° pulse length was adjusted to about 14.09 μs for each sample. Moreover, J-resolved spectroscopy, COSY, HMBC, and HSQC experiments were acquired for the signal attribution of metabolites.

The ^1^H-NMR spectra were imported to the MestReNova 9.0.1 software for phase and baseline correction and zero filling. The data were normalized through the total area to convenient compared of samples among groups, and the ^1^H-NMR spectra were segmented and integrated with a width of 0.003 ppm. After normalization and Pareto scaling, the data matrix was imported into SIMCA^®^ (Version 13.0, Umetrics, Sweden) to perform multivariate statistical analysis, mainly for principal component analysis (PCA) and orthogonal partial least squares discriminant analysis (OPLS-DA). The differential metabolites were screened based on the correlation coefficient of the spectra in the loading score plot (r > 0.6 was considered a significantly different metabolite) as previously described (Wang et al., 2022).

### 2.7 Hepatic lipidomics analysis

The liver tissue (100 mg) extracted with 435 μl of precooled water was fully homogenized by using a rapid tissue cell disrupter (FLUKO-F8, Shanghai, China) on dry ice, and then a total of 1.5 ml precooled dichloromethane:methanol (2:1) was added to the tissue for extraction and subjected to centrifugation (2,500 × *g*, 4°C, 15 min). Repeat that above steps to combine the supernatant after the two extractions, drying with nitrogen, dried lipid extracts were re-suspended in 200 μl of 2-propanol/methanol (1:1, *v/v*). A total of 20 μl of supernatant was transferred into the LC/MS vial for analysis. A quality control (QC) sample was prepared by pooling equal volumes of all samples for analysis.

The chemical components were detected using the Q Exactive™ Q-Orbitrap™ Mass Spectrometer coupled with the UltiMate® 3000 UHPLC system. Chromatographic separation was achieved on Hypersil GOLD™ C18 (100 × 2.1 mm, 1.9 μm; Waters) maintained at 45°C using a binary mobile phase which consisted of acetonitrile:water (60:40, *v/v*) solution containing 5 mM ammonium formate and 0.1% formic acid (A) and acetonitrile:2-propanol (10:90, *v/v*) solution containing 5 mM ammonium formate and 0.1% formic acid (B). The mobile phase had a flow rate of 0.35 ml/min according to an optimal gradient eluting program: 0–14.5 min 40%–100% (B), 14.5–16.5 min 100% (B), 16.50–16.51 min 100–40% (B), and 16.51–20 min 40% (B). An injection volume of 4 µl was set. High-resolution MS data were recorded on a Q Exactive™ Quadrupole-Orbitrap™ Mass Spectrometer in the negative ESI mode. The source parameters were spray voltage, −3.5 kV/+2.8 kV; capillary temperature, 350°C; gas temperature, 350°C; sheath gas flow rate, 35 arb; auxiliary gas flow rate: 15 arb; and sweep gas flow rate, 1 arb. The Orbitrap analyzer scanned over m/z 250–1500 at a resolution of 70,000 in full-scan MS^1^ and at 17,500 in dd-MS^2^. To obtain more balanced MS^2^ spectra for ginsenosides with different sugars, mixed normalized collision energies (MNCE) at 15, 25, and 35 V were adopted. Data acquisition and processing were controlled and performed by using the Thermo Xcalibur™ 4.1 software.

## 3 Results

### 3.1 Chemical composition

**FIGURE 1:**
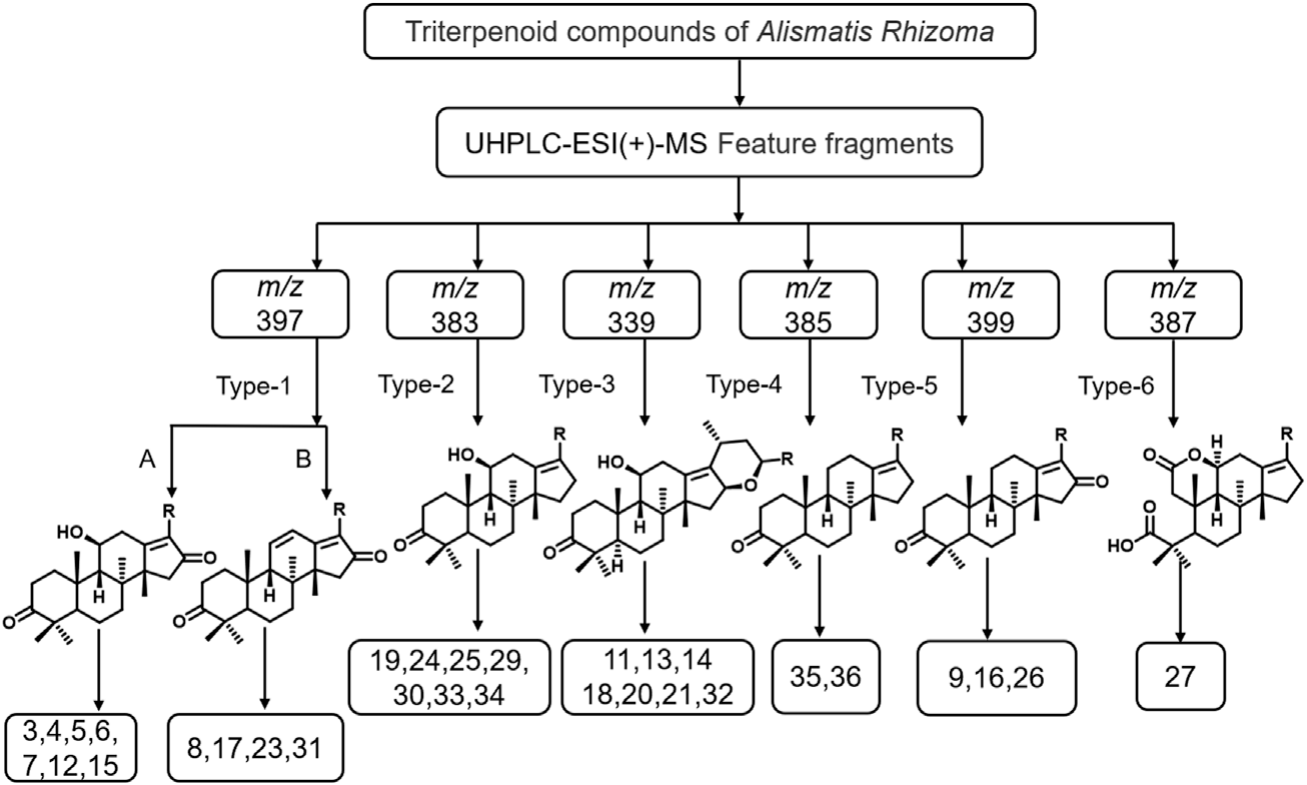
The mass spectrometric characteristic fragment types of triterpenoids from Alismatis Rhizoma extract.

The chemical components in Alismatis rhizoma were analyzed using the Thermo Xcalibur™ 4.1 software. As the triterpenoids and sesquiterpenoids, found rich in Alismatis rhizoma, exhibit high responses in the positive ion mode, the positive ion mode detection was mainly adopted. Based on the data reported in the relevant literature, a total of 31 triterpenoids and 2 sesquiterpenoids in the extract of Alismatis rhizoma were identified (Gao et al., 2018; Yang et al., 2019). By comparing with the standard substance, seven compounds were confirmed and are given in the Supplementary Table S1 of the supplementary material, and according to the identification results, we summarized the six main types of characteristic fragments in the Alismatis rhizoma extract, and the results are shown in Figure 1.

The identification results showed that triterpenoids are the main components in the Alismatis rhizoma extract. According to the mass spectrometric identification results of the Alismatis rhizoma extract, the mass spectral fragmentation pattern of the 31 triterpenoids from the Alismatis rhizoma extract are summarized. The ring opening of the branch chain of alisol B-23 acetyl ester was confirmed to form alisol A-23 acetyl ester, thus the identified triterpenoids mainly took alisol A as the basic mother nucleus. At the same time, the acetyl group was easily substituted at C-11, C-23, and C-24, and the carbonyl group was easily substituted at C-11, C-16, and C-7. A three-membered oxygen ring was easily formed at positions 13 and 17; a three-membered oxygen ring was easily formed at positions C-24 and C-25; a six-membered oxygen ring was easily formed at C-16 and C-23; and a five-membered oxygen spiro ring was easily formed at C-23 and C-17 as given in Supplementary Figure S2 of the supplementary material.

### 3.2 Biochemical analysis and histopathology

During the 9 weeks, the trend of body weight change is shown in the supplementary materials in Supplementary Figure S3A. When compared to the control group, the body weight of the mice in the model group kept increasing with significant difference. The changes in the fasting and postprandial blood glucose values of mice at the 13th week are shown in the supplementary materials in Supplementary Figure S3B. When compared to the other groups, the postprandial blood glucose (PBG) value in the model group was significantly increased and the blood glucose values in the administration group were significantly decreased. In addition, the fasting blood glucose (FBG) values in the model group were decreased, while those of postprandial blood glucose were increased, which was probably due to insufficient insulin secretion after meals, leading to an increase in blood glucose. However, in the absence of food intake, the insulin secretion was relatively sufficient, such that the fasting blood glucose did not rise to a certain extent. In this study, the results have shown that the administration group had a certain hypoglycemic effect when compared with the model group.

**FIGURE 2:**
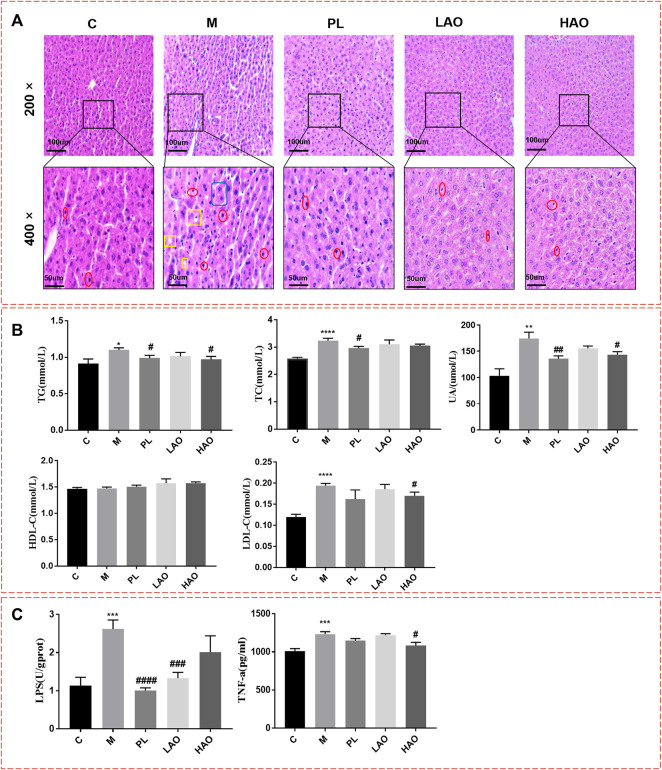
Mice liver tissue section and biochemical indexes. **(A)** H&E staining diagram of pathological section of liver of mice in each group with magnification of 200× and 400× (where the position marked with red represents inflammatory cell infiltration; Yellow represents fatty degeneration and the generation of fat vacuoles; Blue represent hepatomegaly); **(B)** Statistical graph of serum biochemical indexes; **(C)** Statistical chart of liver biochemical indicators (*p < 0.05, **p < 0.01, ***p < 0.001 and ****p < 0.001, vs C; #*p* < 0.05, ##*p* < 0.01, ###*p* < 0.001 and ####*p* < 0.0001, vs M).

Histological analysis of the H&E stained tissue sections (Figure 2A) suggests that the liver tissue structure of the control group was normal and the cell structure was intact and neatly arranged. In the model group, the cells around the hepatic lobule were obviously swollen, with obvious lymphocyte infiltration and hepatocyte necrosis. No scattered vacuoles generated by steatosis were observed in the liver of the treatment group, and the structure of the hepatic lobules was clearer than that of the model group. The results of the serum biochemical analysis (Figure 2B) indicated that when compared with the control group, the levels of TG, TC, LDL-C, and UA in the model group were significantly increased, while the levels of TG, TC, LDL-C, and UA in the pioglitazone and Alismatis rhizoma extract treatment groups were markedly decreased (*p* < 0.05). The results of liver ELISA (Figure 2C) show that as compared to the control group, the levels of LPS and TNF-α in the model group were notably increased, while those in the pioglitazone and Alismatis rhizoma extract treatment groups tended to have decreased. The results of the serum biochemical analysis showed that the levels of TG, TC, LDL-C, and UA after the pioglitazone and Alismatis rhizoma extract treatments were markedly decreased.

According to the body weight and blood glucose change trend of the mice, the test results of the biochemical indicators and H&E staining in the control group, model group, and the dosing treatment group all suggest that high-fructose drinks caused metabolic syndrome, and the Alismatis rhizoma extract had a damage-reducing effect .

### 3.3 Metabolomics analysis

The signal assignments of the typical ^1^H-NMR spectra of the liver tissue are shown in Supplementary Figure S4A. The ^1^H-NMR spectrum signal is elucidated from the literature (Wu et al., 2020b) and the Human Metabolome Database (https://hmdb.ca/) and is further confirmed by two-dimensional NMR data. A total of 52 metabolites were identified from the liver tissue, that mainly included amino acids, organic acids, alkaloids, sugars, and nucleotides (Supplementary Table S2).

**FIGURE 3:**
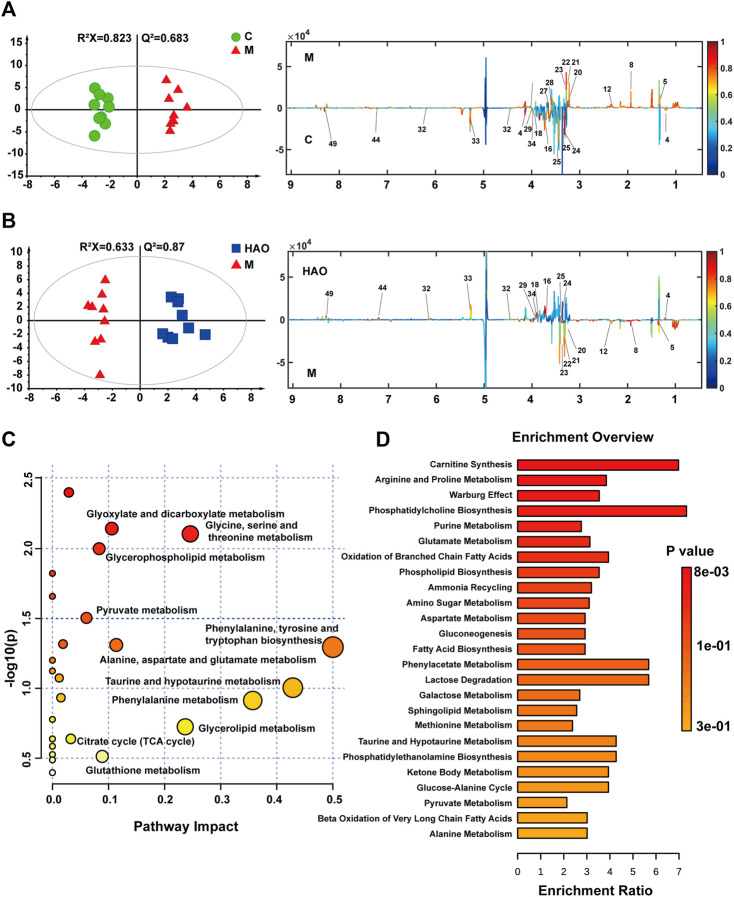
OPLS-DA score plots (left) and the corresponding loading (right) plots derived from 1H NMR spectra of liver tissue **(A,B)**. Analysis of metabolic pathways related to high fructose-induced metabolic syndrome **(C)**. The pathway enrichment analysis based on 1H-NMR metabolomics of liver tissue **(D)**.

**FIGURE 4:**
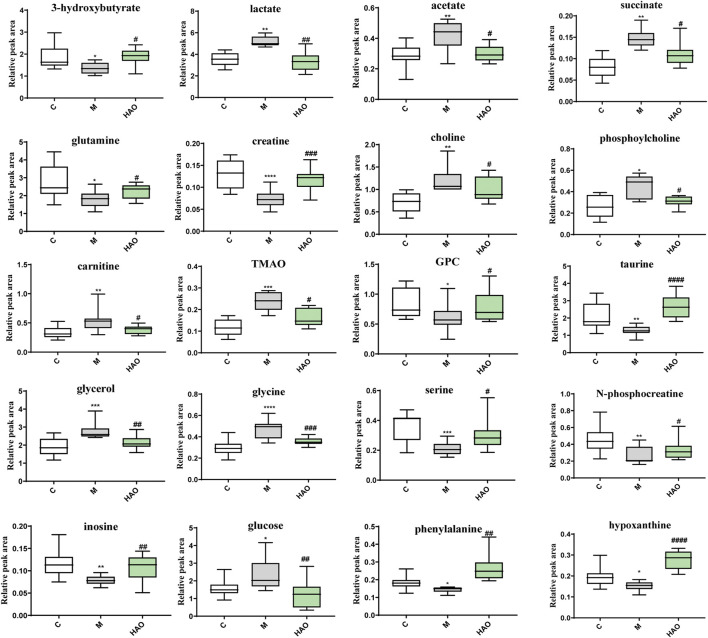
The levels of differential metabolites based on 1H-NMR analysis of liver tissue. (*p < 0.05, **p < 0.01, ***p < 0.001 and ****p < 0.001 vs C group; #*p* < 0.05, ##*p* < 0.01, ###*p* < 0.001 and ####*p* < 0.0001 vs M group).

After pairwise comparison (the control group vs the model group, the HAO group vs the model group, the LAO group vs the model group.), it was found that the clustering effect of the HAO group was better than that of the LAO group. In order to better study the potential biomarkers of the Alismatis rhizoma extract on high-fructose–induced metabolic syndrome, we selected the HAO group for further metabolomics analysis. The results of the principal component analysis (PCA) suggested that the liver tissue samples of the control group, model groups, and the Alismatis rhizoma extract group were significantly separated (Supplementary Figure S4B), indicating that the endogenous metabolites in each group had significant differences. To investigate the difference in the metabolic spectra in the case of the control group *vs*. the model groups and the high-dose groups *vs*. the model groups, the ^1^H-NMR data matrix was placed in a supervised OPLS-DA model (Figure 3). The study results showed that there was an obvious separation; meanwhile, the permutation analysis of the cross-validation 200 times indicated that there was no overfitting of the OPLS-DA model (Supplementary Figure S4A). The change of color of the spectral signals from blue to red in different groups of the loading plot indicated that the correlation coefficient was getting larger and larger, and a correlation coefficient greater than 0.6 was considered to be a significant differential metabolite (Figures 3A,B). A total of 20 differential metabolites of high-fructose–induced metabolic disorder in mice were screened as shown in Figure 4. The levels of lactate, acetate, succinate, choline, phosphorylcholine, carnitine, TMAO, glycerol, glucose, and glycine obviously increased when compared with those of the model groups, and the content levels were significantly improved after the treatment with the Alismatis rhizoma extract. In addition, it is noteworthy that the model groups had downregulated trends of 3-hydroxybutate, glutamine, creatine, GPC, taurine, serine, N-phosphate, inosine, phenylalanine, and hypoxanthine than did the control group, whereas these were found increased after the Alismatis rhizoma extract treatment (Figure 4 and Supplementary Table S3). According to the pathway analysis function of the MetaboAnalyst 4.0 online platform (https://www.metaboanalyst.ca/), it was found that a total of 20 differential metabolites were involved in 11 metabolic pathways, namely, glycine, serine, threonine, glycolysis, lipid (glycerophospholipid and glycerolipid), taurine and hypotaurine, energy (pyruvate and TCA cycle), phenylalanine, phenylalanine, tyrosine and tryptophan, alanine, aspartate and glutamate, glutathione, and glyoxylate and dicarboxylate metabolisms as shown in Figure 3C.

The KEGG metabolic pathway enrichment analysis of the 20 differential metabolites is based on ^1^H-NMR metabolomics analysis of the liver tissue as shown in Figure 3D. It could be determined that the Alismatis rhizoma extract affected carnitine synthesis, arginine and proline metabolisms, oxidation of branched-chain fatty acids, phospholipid biosynthesis, sphingolipid metabolism, taurine and hypotaurine metabolisms, amino sugar metabolism, alanine metabolism, glutathione metabolism, and glutamate metabolism.

### 3.4 Lipidomics analysis

The data matrix that included all groups was obtained using the MS-DIAL 4.7 software, and SIMCA 14.1 was used for multivariate statistical analysis (Supplementary Figure S5). The PCA score plot suggested that there were significant differences among the control, model, and HAO groups in the liver (Supplementary Figure S5A). According to the OPLS-DA results (Supplementary Figure S5B–E), it was found that the clusters among the control, model, and HAO groups showed good and significant separations. These results indicate that the Alismatis rhizoma extract could significantly improve fructose-induced metabolic syndrome.

**FIGURE 5:**
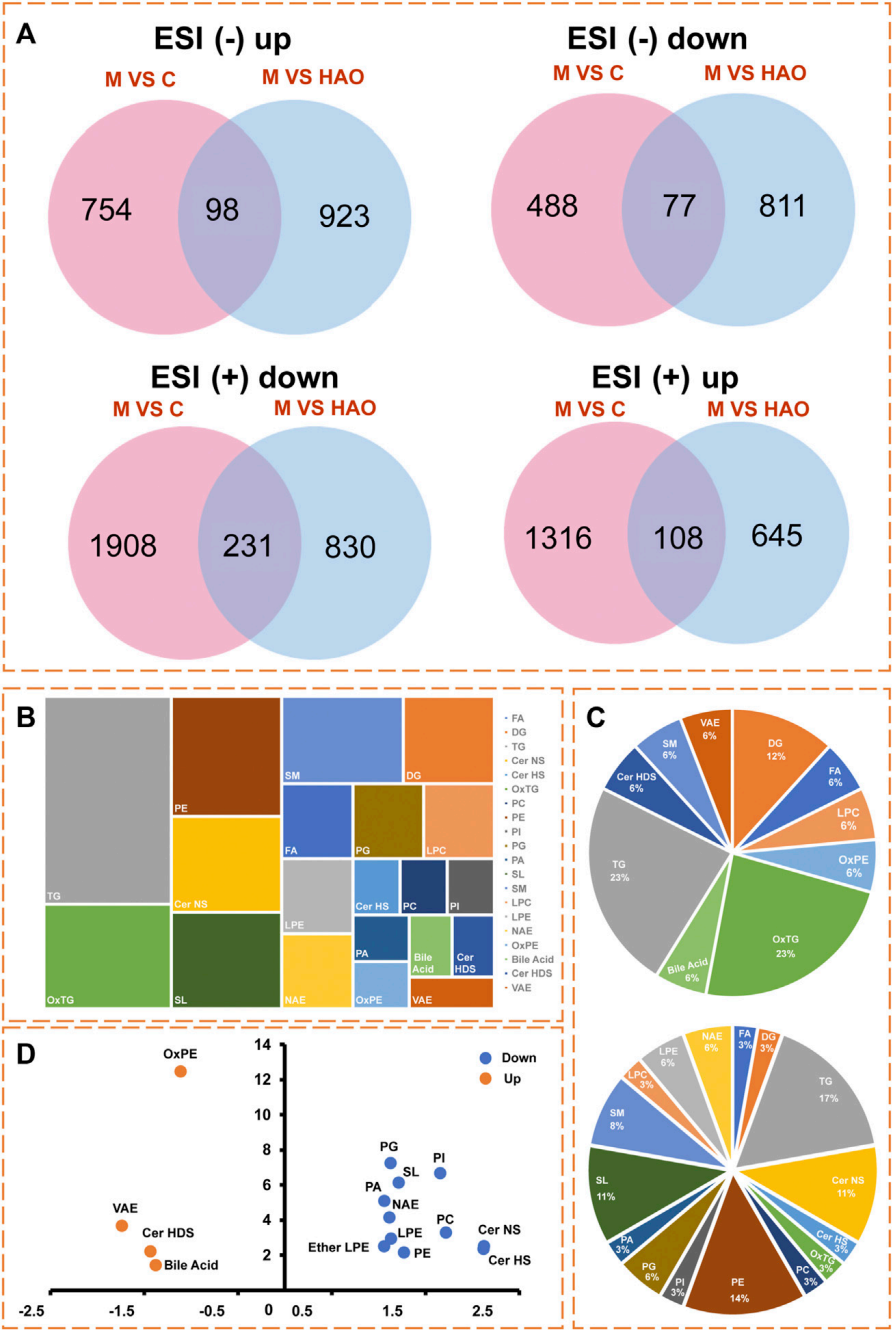
The differential lipid analysis of liver tissue based on the UHPLC-Q/Orbitrap MS. **(A)** Venn analysis of differential lipids in different ion modes. **(B)** The type analysis of 53 differential lipid compounds in lipidomics. **(C)** Pie chart of the proportion of 17 upregulated lipid compounds, and Pie chart of the proportion of 36 downregulated lipid compounds. **(D)** Lipid types that present an up-regulated or down‐regulated trend.

In order to obtain the Alismatis rhizoma extract to improve the differential lipids of fructose-induced metabolic syndrome, two screening conditions (FC > 2 and *p* < 0.05) were set to screen the data at the same time as shown in Figure 5A. The Venn analysis was performed on the compounds in different scanning modes, and the results suggested that there were 108 compounds that were upregulated and 231 compounds that were downregulated in the positive ion mode, while 98 compounds were upregulated and 77 compounds were downregulated in the negative ion mode after treatment with the Alismatis rhizoma extract. According to the accurate molecular ion masses, the retention times, MS-DIAL database (http://prime.psc.riken.jp/compms/msdial/main.html), LIPID MAPS database (https://www.lipidmaps.org/), and HMDB database (https://hmdb.ca/), further identification of the common compounds from the Venn analysis in different scanning modes was performed and a total of 53 differential compounds were identified, which included 36 downregulated lipid compounds and 17 upregulated lipid compounds as listed in Supplementary Table S4.

Through lipidomics data analysis as shown in Figure 5B, it was found that a total of 17 upregulated lipid compounds belonged to bile acid, Cer HDS, DG, LPC, FA, OxPE, OxTG, SM, TG, and VAE as shown in Figure 5C, while 36 downregulated compounds out of the 53 differential compounds belonged to Cer HS, Cer NS, DG, LPE, NAE, OxTG, PC, PE, SM, Ether LPE, LPC, SL, FA, PA, PG, and PI as shown in Figure 5C. Among these, bile acid, Cer HDS, OxPE, and VAE lipids showed an upregulated trend, whereas the Cer HS, Cer NS, LPE, NAE, PC, PE, Ether LPE, SL, PA, PG, and PI types showed a downregulated trend as shown in Figure 5D.

### 3.5 Metabolic pathway analysis

**FIGURE 6:**
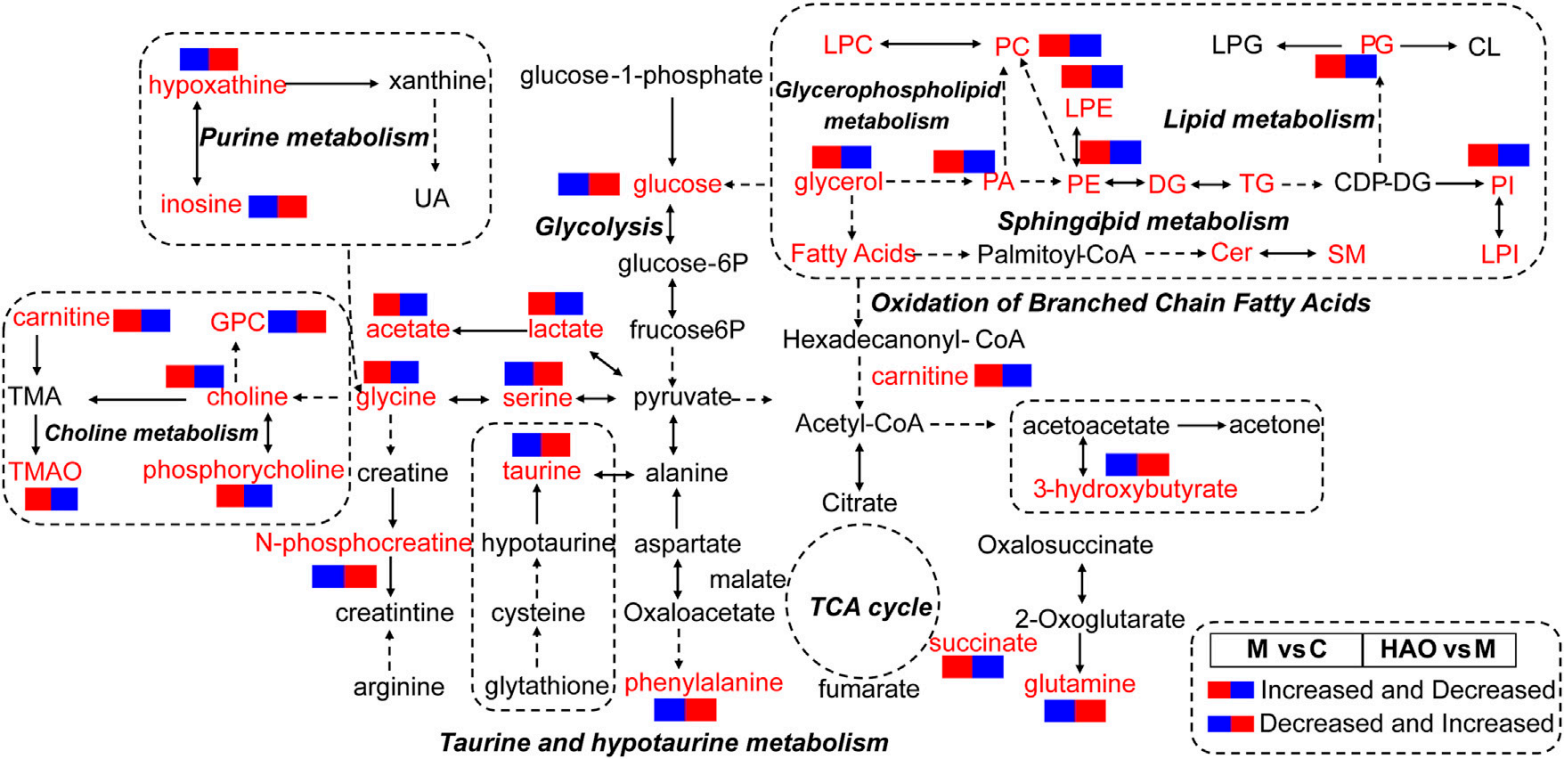
The analysis of metabolic pathway of Alismatis Rhizoma extract in intervention of high fructose-induced metabolic syndrome in mice. (The red font represented the detected differential metabolites, the solid arrow represented one-step production, and the dotted arrow represented multiple-step production.).

Based on the results of metabolomics and lipidomics studies, it was concluded that the Alismatis rhizoma extract could regulate high-fructose–induced metabolic syndrome and return it to a normal level by affecting purine metabolism, glycolysis, choline metabolism, taurine and hypotaurine metabolisms, and phospholipid metabolism. According to the metabolomics and lipidomics profiling in the liver, the metabolic network of potential differential metabolites is shown in Figure 6.

## 4 Discussion

The Alismatis rhizoma extract has been identified as mainly triterpenoid compounds by high-resolution mass spectrometry, and the triterpenoids from the Alismatis rhizoma extract can alleviate metabolic syndrome caused by high-fructose drinks. The metabolic syndrome has exploded in recent years as a result of excessive intake of fructose due to lifestyle changes and excessive pursuit of food taste. In the face of this situation, in addition to routine medical treatment, the use of natural plants for prevention and symptom reduction is also very important. In this study, the chemical components of the Alismatis rhizoma extract were analyzed by using UHPLC-Q/Orbitrap MS, and a total of 31 triterpenoids and 2 sesquiterpenoids were identified. It was found that the main chemical components of the Alismatis rhizoma extract were triterpenoids. In addition to this, the types of triterpenoids were summarized, which further clarified the pharmacodynamic material basis of the Alismatis rhizoma extract and provided the experimental and theoretical material basis for the treatment of fructose-induced metabolic syndrome with the Alismatis rhizoma extract.

Metabolomics suggested that that Alismatis rhizoma extract could reduce the amino acid metabolism disorder and choline metabolism disorder caused by high-fructose drinks, improve the content of glutathione, and accelerate glycolysis and purine metabolism *in vivo*. Clinical studies have shown that since fructose bypasses the rate-limiting step catalyzed by phosphofructokinase in the liver, resulting in a high burden on *de novo* lipogenesis and uric acid production, a large intake of fructose can induce metabolic syndrome (Lima et al., 2015). After analyzing the metabolome data, it has been clarified that fructose not only causes changes in 32 components, such as TMAO, after entering mice but also affects 17 metabolic pathways, such as glutathione metabolism, through pathway enrichment analysis. Based on previous literature, the increased level of TMAO *in vivo* leads to cardiovascular diseases closely related to metabolic syndromes, such as atherosclerosis, coronary heart disease, and type II diabetes (He et al., 2021). The treatment groups could significantly downregulate the levels of glycerol, glucose, and TMAO and increase the content of taurine and antioxidants *in vivo* by regulating the glutathione pathway, indicating that the Alismatis rhizoma extract–administrated groups could improve the disorder levels of the metabolites *in vivo* to treat high-fructose–induced metabolic syndrome.

Lipidomics suggests that the Alismatis rhizoma extract could increase bile acid content, reduce lipid accumulation, especially phospholipid lipids, and improve lipid metabolism disorders *in vivo*. It has been reported that fatty acid amide hydrolase (FAAH) degrades two major lipid metabolites, N-acyl ethanolamine (NAE) and N-acyl taurines (NATs), which act as signaling molecules in the central nervous system and peripheral tissues. The increased contents of the NAE and NATs will lead to mice developing susceptibility to obesity, fatty liver, and insulin resistance phenotypes (Drin, 2014). The two most abundant glycerophospholipids, PC and PE, in animal tissues were metabolized by phospholipases to arachidonic acid and LPC and LPE. PC and PE play a key role in inflammation as the key precursors of proinflammatory lipids that affect the physical properties and functional integrity of the membranes (Dennis and Norris, 2015). The accumulation of PC and PE can cause hepatocellular dysfunction, leading to hepatocellular apoptosis, inflammation, and the progression of liver diseases (Wu et al., 2019). The accumulation of LPC induces hepatocyte lipid apoptosis (Kakisaka et al., 2012), leading to mitochondrial dysfunction (Hollie et al., 2014), and induces the release of fibroblast-like extracellular vesicles (EV) by hepatocytes (Ibrahim et al., 2016), which indicates an increase in liver LPC content in nonalcoholic steatohepatitis (NASH) accompanied by an increase in the severity of liver disease (Puri et al., 2007; Zhou et al., 2016). Changes in PI in plasma and liver patients have been associated with cirrhosis (Buechler and Aslanidis, 2020) and hepatocellular carcinoma (HCC) (Li et al., 2017). Wu et al. (2020b) confirmed by using lipidomics that PC, LPC, PE, LPE, and PI were the most increased lipids in the liver injury group. Furthermore, bile acids could regulate metabolic homeostasis by completing the transformation of primary bile acids into secondary bile acids in the intestine–liver axis to achieve the effects of regulating blood glucose and lipids in preventing obesity (Chiang and Ferrell, 2020). In this study, the treatment group could significantly downregulate the contents of PC, PE, PI, PG, PA, LPE, Cer NS, Cer HS, and NAE and upregulate the contents of bile acid to reduce the accumulation of lipids, indicating that the Alismatis rhizoma extract–administered groups could improve the disorder level of lipids *in vivo* to treat high-fructose–induced metabolic syndrome.

Metabolomics and lipidomics complement each other. Their results have shown that a high-fructose diet could lead to metabolic disorders of branched-chain amino acids (that mainly include leucine and isoleucine valine), which not only reduced the contents of cysteine and glutamic acid but also led to the reduction of GSH, while leading to branched alpha-keto acid (BCKA, direct catabolites of BCAAs), directly inhibiting the TCA cycle, adding metabolites such as pyruvic acid and lactic acid, and inhibiting energy metabolism, leading to the occurrence of obesity and insulin resistance (Roberts et al., 2020). After the Alismatis rhizoma extract, which mainly contains triterpenoid components, was administered for treatment, the synthesis of glutamic acid was accelerated, such that the content of taurine and hypotaurine was increased, and the synthesis of bile acid could be accelerated by taurine. The generated bile acid could reduce the generation of lipids, reduce the accumulation of lipids *in vivo*, and accelerate the energy metabolism *in vivo*.

At the same time, through metabolomics and lipidomics analysis, the synthesis of serine and alanine was accelerated in order to reduce the pyruvic acid content *in vivo*, and TMAO, choline, and uric acid were reduced. The Alismatis rhizoma extract could not only regulate purine metabolism and reduce uric acid (by alisol B 23-acetate, alisol B, alisol A 24-acetate, alisol A, and alisol C 23-acetate) (Zhang et al., 2017b) but also regulate lipid metabolism, phospholipid metabolism (by alisol A 24-acetate, alisol B 23-acetate, alisol F, and alismol) (Choi et al., 2019), glycolysis, choline metabolism, and taurine and hypotaurine metabolisms (by 16-oxo-alisol A, 16-oxo-alisol A 23-acetate, 16-oxo-alisol A 24-acetate, alisol C, alisol C 23-acetate, alisol L, alisol A, alisol A 23-acetate, alisol A 24-acetate, alisol L 23-acetate, alisol B, alisol B 23-acetate, 11-deoxy-alisol B, and 11-deoxy-alisol B 23-acetate) (Jia et al., 2021), which is consistent with previous reports.

## 5 Conclusion

In this study, the structural characteristics of triterpenoids in the Alismatis rhizoma extract were clarified through the identification of compounds in the extract of Alismatis rhizoma. Moreover, the therapeutic effect and potential mechanism of the Alismatis rhizoma extract on high-fructose–induced metabolic syndrome were elucidated from the perspective of metabolomics and lipidomics, which were mainly related to purine metabolism, glycolysis, choline metabolism, taurine and taurine metabolism, and lipid metabolism. To sum up, this study suggests that Alismatis rhizoma extract may have a good therapeutic effect on high-fructose–induced metabolic syndrome by improving a series of biochemical indicators, inhibiting energy metabolism, amino acid metabolism, and regulating bile acid to reduce phospholipid content. It provides a strategy and approach for natural plants to treat metabolic syndrome. However, the triterpenes in the Alismatis rhizoma extract affect the target *in vivo*, which needs to be further clarified, and how to make a dosage form for the clinical treatment of metabolic syndrome also needs further consideration.

## Data Availability

The original contributions presented in the study are included in the article/Supplementary Material, and further inquiries can be directed to the corresponding author.

## Abbreviations

Cer HS, ceramide hydroxy fatty acid sphingosine; Cer HDS, ceramide hydroxy fatty acid dihydrosphingosine; Cer NS, ceramide non-hydroxy fatty acid sphingosine; DG, diglyceride; Ether LPE, ether lysophosphatidylethanolamine; FA, fatty acid; HDL-C, high-density lipoprotein cholesterol; LDL-C, low-density lipoprotein cholesterol; LPC, lysophosphatidylcholine; LPE, lysophosphatidylethanolamine; NAE, N-acyl ethanolamine; Q-Orbitrap-MS, quadrupole–Orbitrap–mass spectroscopy; OxPE, oxidized phosphatidylethanolamine; OxTG, oxidized triglycerides; PA, phosphatidic acid; PC, phosphatidylcholine; PI, phosphatidylinositol; PE, phosphatidylethanolamine; SL, saccharolipids; SM, sphingomyelin; serum TC, cholesterol; TG, triglyceride; TMAO, trimethylamine oxide; UHPLC, ultra-high performance liquid chromatography; VAE, vitamin A fatty acid ester.
