# Supplementary material for: Alismatis Rhizoma methanolic extract—Effects on metabolic syndrome and mechanisms of triterpenoids using a metabolomic and lipidomic approach
Source: Front Pharmacol. 2022 Sep 9;13:983428. doi: 10.3389/fphar.2022.983428 (PMC9500195; doi:10.3389/fphar.2022.983428)
Supplement: Supplementary file 1 [file DataSheet1.docx]

**Supplementary Materials**

**Contents**

**Fig.S1** Preparation proces of *Alismatis rhizoma* extract.

**Fig.S2** Conversion between alisol B 23- acetyl ester and alisol A 23- acetyl ester

**Fig.S3** The levels of blood glucose and body weight in mice. A: The weekly body weight changes in mice and body weight measurements at the 13th week (*n*=12, ^*^ *p* < 0.05, ^**^ *p* < 0.01, *vs* C; ^#^ *p* < 0.05, *vs* M); B: Statistical graphs of fasting and postprandial blood glucose after 4 weeks of modeling and 9 weeks of treatment (*n*=12, ^*^ *p* < 0.05, *vs* C; ^#^ *p* < 0.05, ^##^ *p* < 0.01, *vs* M).

**Fig.S4** Signal assignments of typical ^1^H-NMR spectra of liver tissue. A: δ 0.0–9.0 ppm full ^1^H-NMR chemical shift (δ 0.00 ppm TSP-d4 as internal standard); B: a magnified partial view of δ 0.0–3.1 ppm; C: δ 3.0–4.8 ppm, magnified locally; D: Partial enlargement of δ 4.8–9.0 ppm.

**Fig.S5** The Lipidomics analysis based on the UHPLC-Q/Orbitrap MS of liver tissue. A: OPLS-DA score plot of model group and control group, model group and administration group under negative ion mode; B: OPLS-DA score plot of model group and control group, model group and administration group under positive ion mode; C: Venn diagram after pairwise comparison under different scanning modes.

**Table S1** The identification results of chemical components of *Alismatis Rhizoma* extract based on UHPLC-Q/Orbitrap MS

**Table S2** The metabolites of ^1^H-NMR spectra in liver tissues of mice

**Table S3** The differential metabolites based on ^1^H-NMR metabolomics analysis of liver tissue.

**Table S4** The differential lipids result of lipidomics in liver tissue based on UHPLC-Q/Orbitrap MS


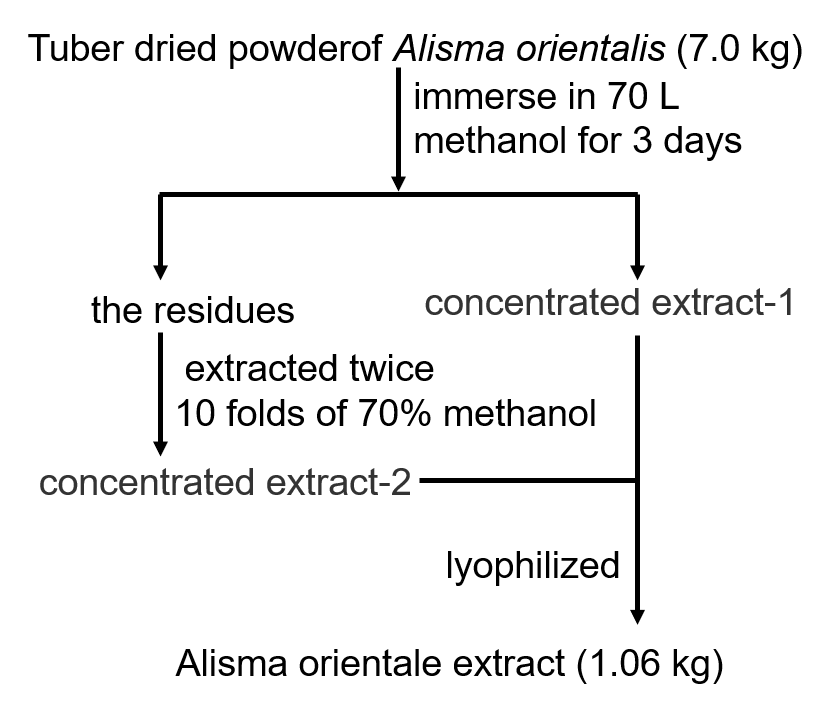


Fig.S1 Preparation proces of Alismatis rhizoma extract.


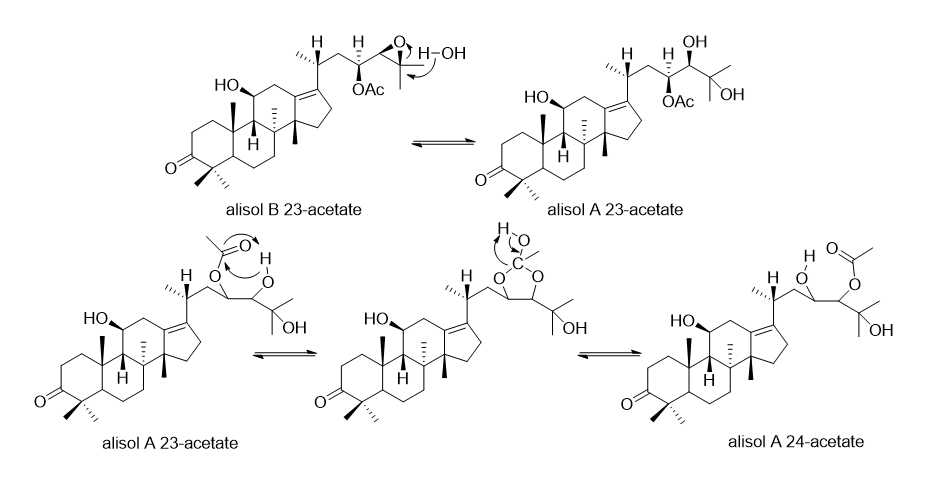


Fig.S2 Conversion between alisol B 23- acetyl ester and alisol A 23- acetyl ester


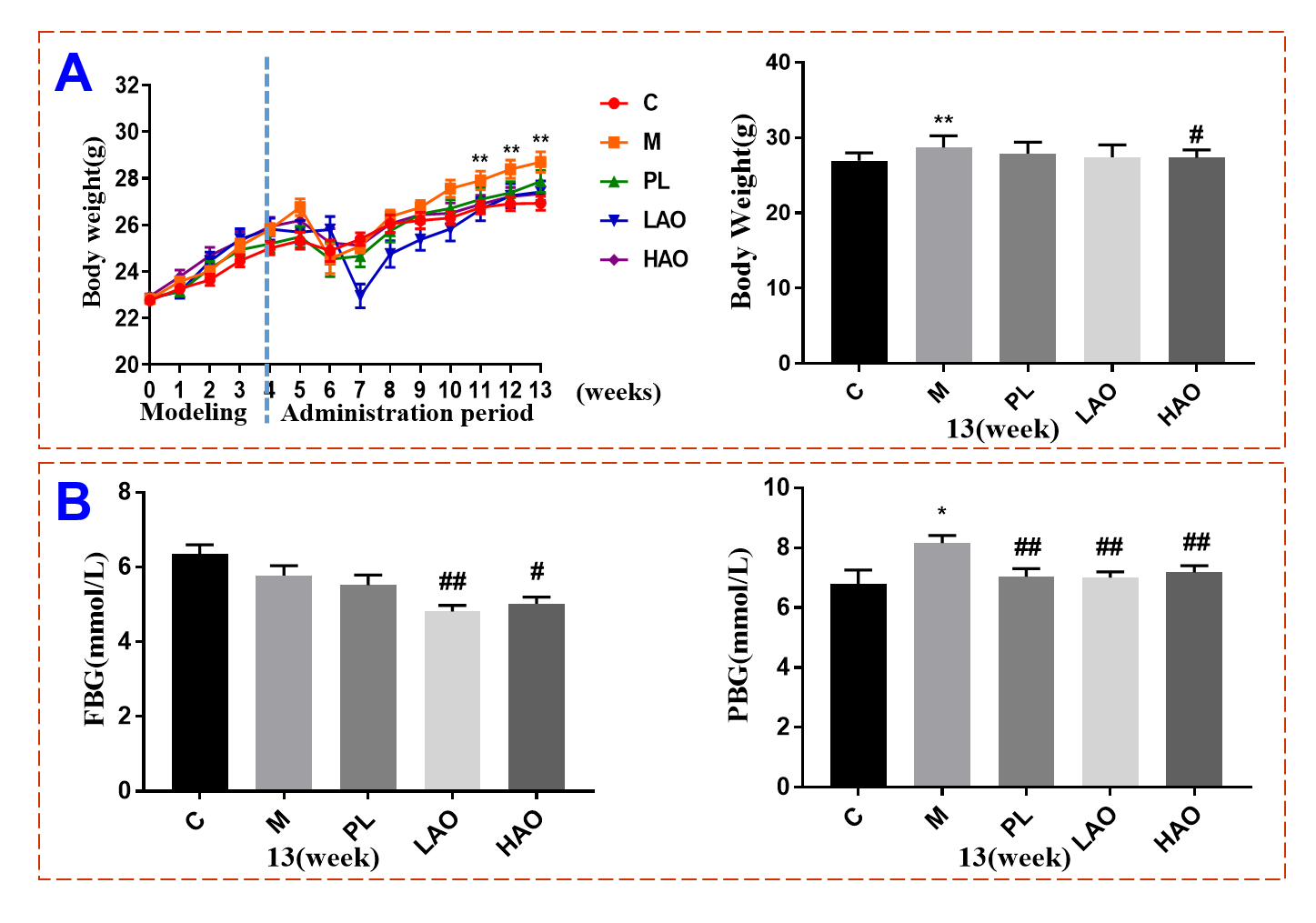


Fig.S3 The levels of blood glucose and body weight in mice. A: The weekly body weight changes in mice and body weight measurements at the 13th week (*n*=12, ^*^ *p* < 0.05, ^**^ *p* < 0.01, *vs* C; ^#^ *p* < 0.05, *vs* M); B: Statistical graphs of fasting and postprandial blood glucose after 4 weeks of modeling and 9 weeks of treatment (*n*=12, ^*^ *p* < 0.05, *vs* C; ^#^ *p* < 0.05, ^##^ *p* < 0.01, *vs* M).


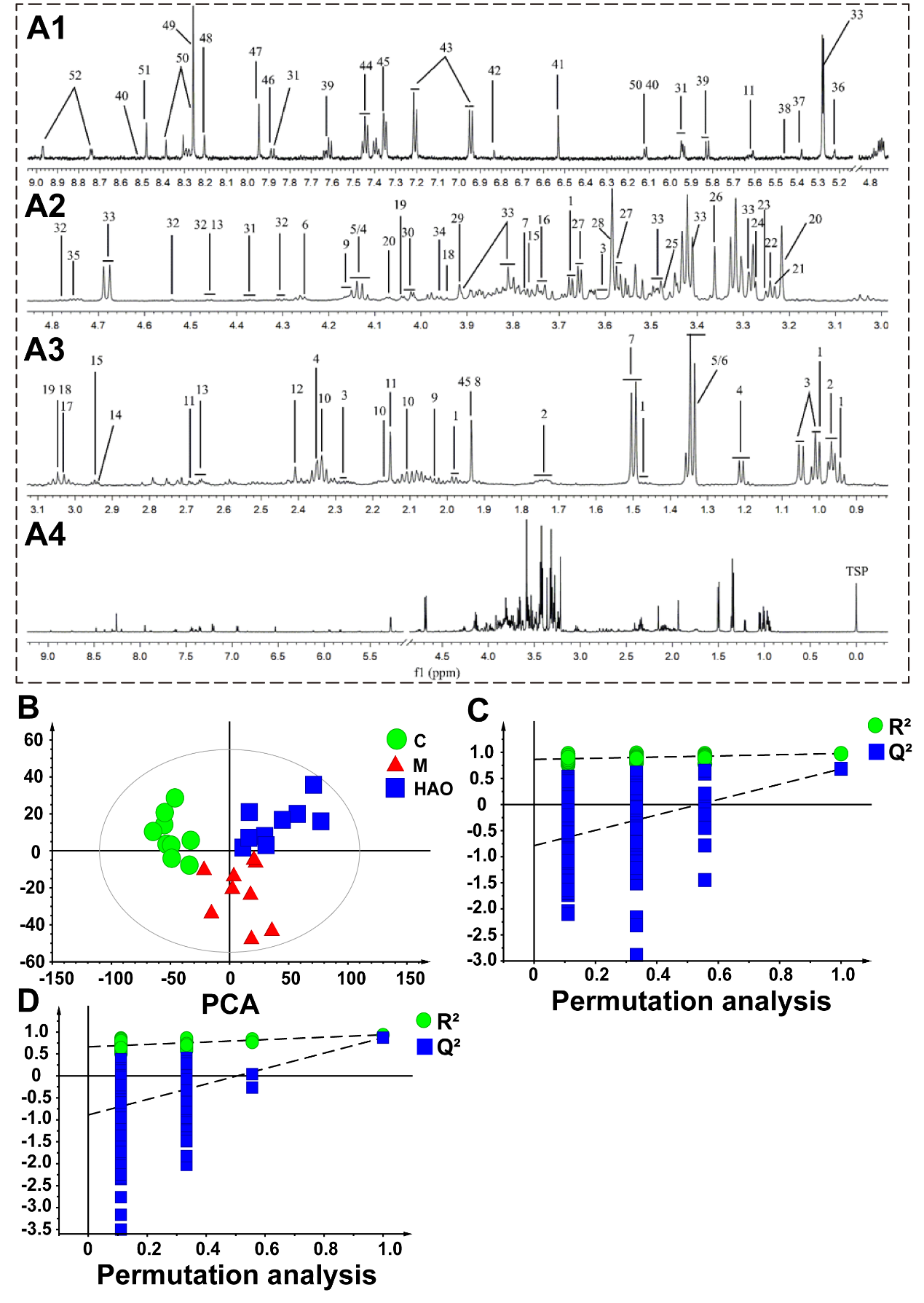


Fig.S4 Signal assignments of typical ^1^H-NMR spectra of liver tissue. A1: Partial enlargement of δ 4.8–9.0 ppm; A2: δ 3.0–4.8 ppm, magnified locally; A3: a magnified partial view of δ 0.0–3.1 ppm; A4: δ 0.0–9.0 ppm full ^1^H-NMR chemical shift (δ 0.00 ppm TSP-d4 as internal standard). B: PCA based on the^1^H NMR of liver tissue. C and D: Permutation analysis of OPLS-DA on M vs C and HAO vs M, respectively.


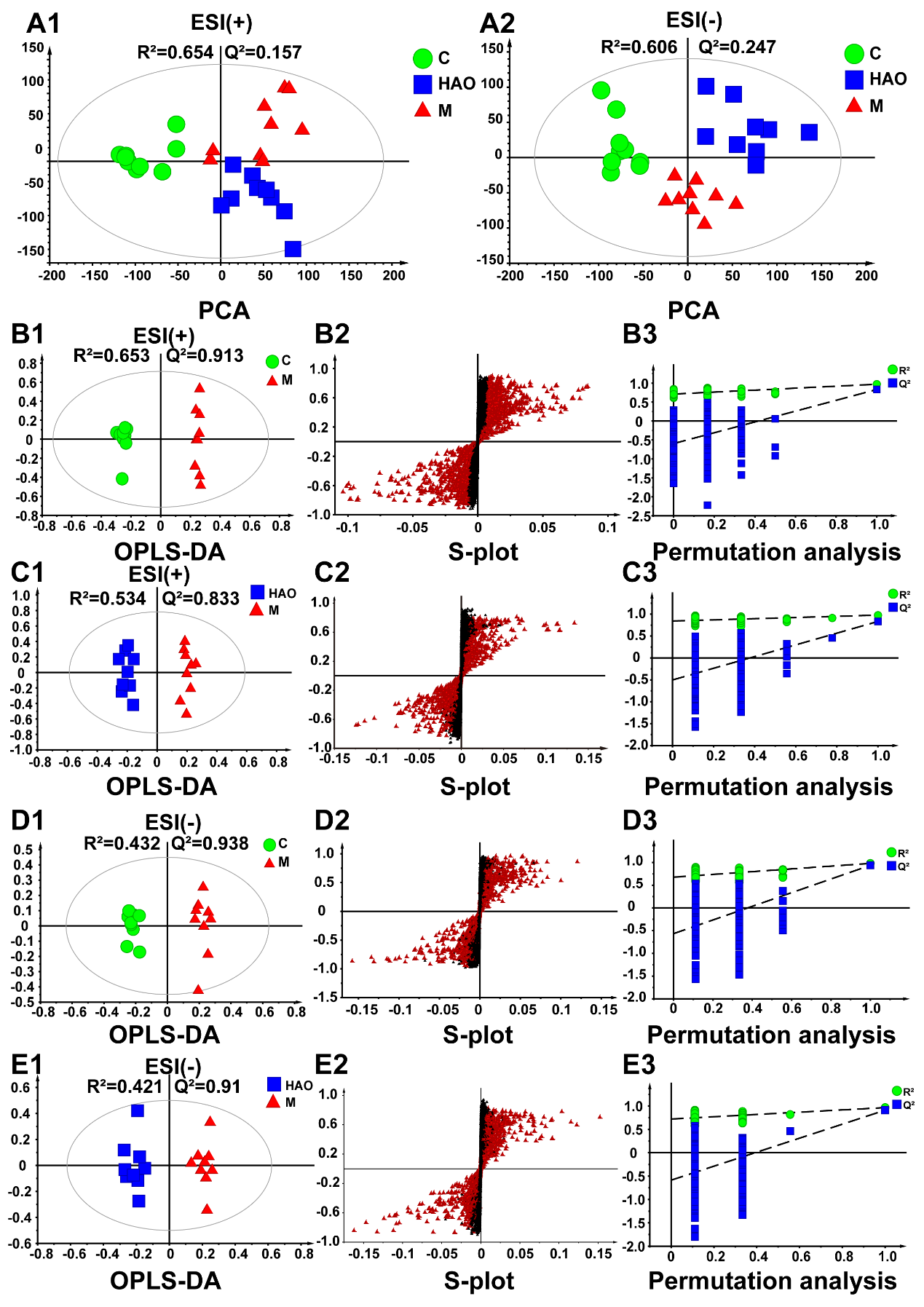


Fig.S5 The Lipidomics analysis based on the UHPLC-Q/Orbitrap MS of liver tissue. A: PCA in the positive ion (A1) and negative ion (A2), respectively; B: OPLS-DA score plot of model group and control group (B1), S-plot (B2), and permutation analysis (B3) in the positive ion; C: OPLS-DA score plot of model group and control group (C1), S-plot (C2), and permutation analysis (C3) in the positive ion; D: OPLS-DA score plot of model group and control group (D1), S-plot (D2), and permutation analysis (D3) in the negative ion; E: OPLS-DA score plot of model group and control group (E1), S-plot (E2), and permutation analysis (E3)in the negative ion.

Table S1 The identification results of chemical components of *Alismatis Rhizoma* extract based on UHPLC-Q/Orbitrap MS

| No. | RT (min) | Mass & adduct | Error (ppm) | ESI-MS^2^ | Formula | Identification |
| --- | --- | --- | --- | --- | --- | --- |
| 1***** | 0.84 | 211.1333 [M+H]^+^ | 2.27 | 211.1333 [M+H]^+^ | C_12_H_18_O_3_ | 1S,4S,10S-calamusin I |
| 2***** | 1.51 | 239.2007 [M+H]^+^ | 0.73 | 221.1897 [M+H-H_2_O]+,  203.1796 [M+H-2H_2_O]+ | C_15_H_26_O_2_ | 1S,4R,5R,10S-guaianediol |
| 3 | 1.94 | 505.3522 [M+H]^+^ | –0.33 | 487.3406 [M+H–H_2_O]^+^,  469.3334 [M+H–2H_2_O]^+^,  451.3187 [M+H–3H_2_O]^+^,  415.2841 [M+H–C_4_H_10_O_2_]^+^,  397.2722 [M+H–C_4_H_10_O_2_–H_2_O]^+^ | C_30_H_48_O_6_ | 16-oxo-alisol A |
| 4 | 2.73 | 547.3629 [M+H]^+^ | –0.02 | 529.3526 [M+H–H_2_O]^+^,  469.3301 [M+H–HAc–H_2_O]^+^,  451.3214 [M+H–HAc–2H_2_O]^+^,  433.3101 [M+H–HAc–3H_2_O]^+^,  415.2831 [M+H–C_6_H_12_O_3_]^+^,  397.2681 [M+H–C_6_H_12_O_3_–H_2_O]^+^ | C_32_H_50_O_7_ | 16-oxo-alisol A 24-acetate |
| 5 | 2.84 | 487.3420 [M+H]^+^ | 0.49 | 469.33.9 [M+H–H_2_O]^+^,  415.2838 [M+H–C_4_H_8_O]^+^,  397.2742 [M+H–C_4_H_10_O_2_]^+^ | C_30_H_46_O_5_ | 25-anhydro-16-oxo-alisol A |
| 6 | 2.94 | 519.3682 [M+H]^+^ | 0.41 | 487.3434 [M+H–CH_4_O]^+^,  469.3313 [M+H–CH_4_O–H_2_O]^+^,  451.3228 [M+H–CH_4_O–2H_2_O]^+^,  415.2835 [M+H–C_5_H_12_O_2_]^+^,  397.2749 [M+H–C_5_H_12_O_2_–H_2_O]^+^ | C_31_H_50_O_6_ | 16-oxo-25-*O*-methyl-alisol C |
| 7 | 3.20 | 487.3418 [M+H]^+^ | 0 | 469.3309 [M+H–H_2_O]^+^,  451.3210 [M+H–2H_2_O]^+^,  415.2846 [M+H–C_4_H_8_O]^+^,  397.2732 [M+H–H_2_O–C_4_H_8_O]^+^ | C_30_H_46_O_5_ | alisol C |
| 8 | 3.63 | 487.3445 [M+H]^+^ | 0.06 | 469.3313 [M+H–H_2_O]^+^,  451.3176 [M+H–2H_2_O]^+^,  397.2739 [M+H–C_4_H_8_O]^+^ | C_30_H_46_O_5_ | 16-oxo-11-anhydro-alisol A |
| 9 | 3.67 | 489.3567 [M+H]^+^ | –1.62 | 471.3473 [M+H–H_2_O]^+^,  453.3312 [M+H–2H_2_O]^+^,  399.2895 [M+H–C_4_H_10_O_2_]^+^ | C_30_H_48_O_5_ | 16-oxo-11-deoxy-alisol A |
| 10 | 4.22 | 513.3574 [M+H]^+^ | –1.25 | 495.3471 [M+H–H_2_O]^+^,  453.3281 [M+H–HAc]^+^,  435.3259 [M+H–HAc–H_2_O]^+^,  381.2778 [M+H–HAc–H_2_O–C_4_H_8_O]^+^,  339.2686[M+H–HAc–H_2_O–C_4_H_8_O–C_2_H_2_O]^+^ | C_32_H_48_O_5_ | 11-anhydro-alisol F 24-acetate or alisol O |
| 11 | 4.73 | 487.3415 [M+H]^+^ | –0.64 | 469.3281 [M+H–H_2_O]^+^,  451.3208 [M+H–2H_2_O]^+^,  397.2721 [M+H–C_4_H_8_O]^+^ | C_30_H_46_O_5_ | 16-oxo-25-anhydro-alisol A |
| 12 | 4.78 | 471.3465 [M+H]^+^ | –0.88 | 453.3383 [M+H–H_2_O]^+^,  381.2714 [M+H–C_4_H_8_O–H_2_O]^+^,  339.2681[M+H–C_4_H_8_O–H_2_O–C_2_H_2_O]^+^ | C_30_H_46_O_4_ | 16,23-oxido-alisol B |
| 13***** | 4.78 | 489.3575 [M+H]^+^ | 0.06 | 471.3430[M+H–H_2_O]^+^,  381.2741 [M+H–H_2_O–C_4_H_10_O_2_]^+^,  339.2681[M+H–H_2_O–C_4_H_10_O_2_–C_2_H_2_O]^+^ | C_30_H_48_O_5_ | alisol F |
| 14* | 5.01 | 529.3513 [M+H]^+^ | –1.98 | 511.3417 [M+H–H_2_O]^+^,  469.3299 [M+H–HAc]^+^,  451.3200 [M+H–HAc–H_2_O]^+^,  415.2842 [M+H–C_6_H_10_O_2_]^+^,  397.2732 [M+H–C_6_H_10_O_2_–H_2_O]^+^ | C_32_H_48_O_6_ | alisol C 23-acetate |
| 15 | 5.38 | 471.3470 [M+H]^+^ | 0.28 | 453.3376 [M+H–H_2_O]^+^,  399.2885 [M+H–C_4_H_8_O]^+^ | C_30_H_46_O_4_ | 11-deoxy alisol C |
| 16 | 5.54 | 469.3314 [M+H]^+^ | 0.39 | 451.3208 [M+H–H_2_O]^+^,  397.2732 [M+H–C_4_H_8_O]^+^ | C_30_H_44_O_4_ | alisol L |
| 17***** | 6.08 | 531.3675 [M+H]^+^ | –0.99 | 513.3571 [M+H–H_2_O]^+^,  495.3466 [M+H–2H_2_O]^+^,  381.2780 [M+H–H_2_O–C_6_H_12_O_3_]^+^,  339.2682[M+H–H_2_O–C_6_H_12_O_3_–C_2_H_2_O]^+^ | C_32_H_50_O_6_ | alisol F 24-acetate |
| 18 | 6.32 | 513.3555 [M+Na]^+^ | 0.94 | 491.3741 [M+H]^+^,  473.3615 [M+H–H_2_O]^+^,  455.3507 [M+H–2H_2_O]^+^,  437.3408 [M+H–3H_2_O]^+^,  383.2943 [M+H–C_6_H_10_O_2_]^+^,  339.2670 [M+H–C_6_H_10_O_2_–C_2_H_2_O]^+^ | C_30_H_50_O_5_ | alisol A |
| 19 | 6.80 | 515.3734 [M+H]^+^ | 0.60 | 497.3625 [M+H–H_2_O]^+^,  437.3408 [M+H–H_2_O–C_2_H_4_O_2_]^+^,  339.2694 [M+C_8_H_16_O_4_] | C_32_H_50_O_5_ | 25-deoxy-alisol F 24-acetate |
| 20 | 6.88 | 453.3361 [M+H]^+^ | –0.45 | 435.3242 [M+H–H_2_O]^+^,  381.2776 [M+H–C_4_H_8_O]^+^,  339.2679 [M+H–C_4_H_8_O–C_2_H_2_O]^+^ | C_30_H_44_O_3_ | 11,25-anhydro-alisol F |
| 21 | 7.91 | 529.3530 [M+H]^+^ | 1.26 | 511.3424 [M+H–H_2_O]^+^,  451.3216 [M+H–H_2_O–C_2_H_4_O_2_]^+^,  397.2722 [M+H–H_2_O–C_6_H_10_O_2_]^+^ | C_32_H_48_O_6_ | isomer of 11-anhydro-16-oxo-alisol A 24-acetate |
| 22 | 8.26 | 515.3732 [M+H]^+^ | 0.25 | 497.3623 [M+H–H_2_O]^+^,  437.3416 [M+H–H_2_O–C_2_H_4_O_2_]^+^,  383.2925 [M+H–C_6_H_12_O_3_]^+^,  339.2675 [M+H–C_6_H_12_O_3_–C_2_H_4_O]^+^ | C_32_H_50_O_6_ | isomer of 11-anhydro alisol A 23-acetate |
| 23 | 8.81 | 473.3623 [M+H]^+^ | –0.42 | 455.3497 [M+H–H_2_O]^+^,  437.3409 [M+H–2H_2_O]^+^,  383.2959 [M+H–H_2_O–C_4_H_8_O]^+^,  365.2831 [M+H–2H_2_O–C_4_H_8_O]^+^ | C_30_H_48_O_4_ | alisol G |
| 24 | 9.26 | 513.3575 [M+H]^+^ | –0.90 | 495.3458 [M+H–H_2_O]^+^,  453.3367 [M+H–C_2_H_4_O]^+^,  435.3257 [M+H–H_2_O–C_2_H_4_O]^+^,  399.2896 [M+H–C_6_H_10_O_2_] ^+^ | C_32_H_48_O_5_ | 11-deoxy alisol C 23-acetate |
| 25***** | 9.62 | 545.3470[M+H]^+^ | –1.56 | 485.3255 [M+H–HAc]^+^,  467.3147 [M+H–HAc–H_2_O]^+^,  387.2530 [M+H–C_8_H_14_O_3_]^+^ | C_32_H_48_O_7_ | alismalactone 23-acetate |
| 26 | 9.77 | 455.3521 [M+H]^+^ | 0.24 | 383.2930 [M+H–C_4_H_8_O]^+^,  341.2845 [M+H–C_4_H_8_O–C_2_H_2_O]^+^ | C_30_H_48_O_3_ | alisol I |
| 27 | 9.80 | 473.3628 [M+H]^+^ | 0.60 | 455.3528 [M+H–H_2_O]^+^,  437.3412 [M+H–2H_2_O]^+^,  383.2930 [M+H–C_4_H_10_O_2_]^+^,  339.2684 [M+H–C_4_H_10_O_2_–C_2_H_4_O]^+^ | C_30_H_48_O_4_ | 25-deoxy-alisol A |
| 28 | 10.11 | 511.3421 [M+H]^+^ | 0.53 | 451.3161 [M+H–HAc]^+^,  397.2746 [M+H–C_6_H_10_O_2_]^+^ | C_32_H_46_O_5_ | alisol L 23-acetate |
| 29 | 10.32 | 455.3517 [M+H]^+^ | –0.55 | 437.3420 [M+H–H_2_O]^+^,  383.2949 [M+H–C_4_H_8_O]^+^,  339.2686 [M+H–C_4_H_8_O–C_2_H_4_O]^+^ | C_30_H_46_O_3_ | 11-anhydro-alisol B |
| 30 | 10.44 | 473.3612 [M+H]^+^ | –2.74 | 455.3512 [M+H–H_2_O]^+^,  437.3414 [M+H–2H_2_O]^+^,  383.2955 [M+H–H_2_O–C_4_H_8_O]^+^,  365.2834 [M+H–2H_2_O–C_4_H_8_O]^+^ | C_30_H_48_O_4_ | alisol B |
| 31***** | 13.52 | 515.3716 [M+H]^+^ | –2.84 | 497.3613 [M+H–H_2_O]^+^,  479.3536 [M+H–2H_2_O]^+^,  437.3414 [M+H–H_2_O–C_4_H_8_O]^+^,  383.2956 [M+H–H_2_O–C_6_H_10_O_2_]^+^,  365.2834 [M+H–2H_2_O–C_6_H_10_O_2_]^+^ | C_32_H_50_O_5_ | alisol B 23-acetate |
| 32 | 16.08 | 457.3672 [M+H]^+^ | –0.88 | 439.3567 [M+H–H_2_O]^+^,  421.3459 [M+H–2H_2_O]^+^,  385.3081 [M+H–C_4_H_8_O]^+^,  341.2819 [M+H–C_4_H_8_O–C_2_H_4_O]^+^ | C_30_H_48_O_3_ | 11-deoxy-alisol B |
| 33 | 19.19 | 499.3784 [M+H]^+^ | 0.71 | 439.3573 [M+H–HAc]^+^,  385.3089 [M+H–C_6_H_10_O_2_]^+^,  341.2837 [M+H–C_6_H_10_O_2_–C_2_H_4_O]^+^ | C_32_H_50_O_4_ | 11-deoxy-alisol B  23-acetate |

* Through comparison with the standard compounds. Compound 1and 2 is sesquiterpenoids, compound 3 to 33 is triterpenoids.

Table S2 The metabolites of ^1^H-NMR spectra in liver tissues of mice

| No. | Metabolites | *δ* ^1^H (Chemical shift) | Assignment |
| --- | --- | --- | --- |
| 1 | isoleucine | 0.94(t), 1.00(d), 1.26(m),  1.47(m), 1.97(m), 3.67(d) | *δ*-CH_3_, *γ'*-CH_3_, *γ*-CH_2_,  *β*-CH, *α*-CH |
| 2 | leucine | 0.95(d), 0.97(t),  1.74(m), 3.74(m) | *δ*, *δ'*-CH_3_, *β*-CH_2_, *γ*-CH,  *α*-CH |
| 3 | valine | 1.00(d), 1.05(d),  2.29(m), 3.60(d) | *γ*-CH_3_, *γ’*-CH_3_, *β*-CH,  *α*-CH |
| 4 | 3-hydroxybutyrate | 1.21(d), 2.35(dd),  2.41(dd), 4.15(q) | *γ*-CH_3_, *α*-CH_2_, *β*-CH |
| 5 | lactate | 1.34(d), 4.15(q) | *β*-CH_3_, *α*-CH |
| 6 | threonine | 1.34(d), 4.25(m) | *γ*-CH_3_, *β*-CH |
| 7 | alanine | 1.5(d), 3.78(m) | *β*-CH_3_, *α*-CH |
| 8 | acetate | 1.93(s) | CH_3_ |
| 9 | proline | 2.04(m), 4.17(m) | *β*-CH_2_, *α*-CH |
| 10 | glutamate | 2.11(m), 2.17(m), 2.34(m) | *β*-CH_2_, *γ*-CH_2_, *α*-CH |
| 11 | O-acetylcarnitine | 2.15(s), 2.69(t),  5.61(dd) | 12-O=C-CH_3_, 4-CH_2_,  5-CH |
| 12 | succinate | 2.41(s) | CH_2_ |
| 13 | malate | 2.66(m), 4.46(d) | 5-CH_2_, 2-CH |
| 14 | trimethylamine | 2.93(s) | CH_3_ |
| 15 | glutathione | 2.95(m), 3.78(m) | 2-CH_2_, 4-CH_2_ |
| 16 | glutamine | 2.13(m), 3.75(dd) | *β*-CH_2_, *α*-CH |
| 17 | cadaverine | 3.03(t) | 2(6)-CH_2_ |
| 18 | creatine | 3.05(s), 3.94(s) | CH_3_, CH_2_ |
| 19 | creatinine | 3.05(s), 4.04(s) | -N(CH_3_), 3-CH_2_ |
| 20 | choline | 3.22(s), 4.07(dt) | -N(CH_3_)_3_, CH_2_ |
| 21 | phosphorylcholine | 3.23(s) | -N(CH_3_)_3_ |
| 22 | carnitine | 3.24(s) | -N(CH_3_)_3_ |
| 23 | TMAO | 3.25(s) | -N(CH_3_)_3_ |
| 24 | GPC | 3.27(s) | -N(CH_3_)_3_ |
| 25 | taurine | 3.28(t), 3.42(t) | N-CH_2_, S-CH_2_ |
| 26 | methanol | 3.36(s) | CH_3_ |
| 27 | glycerol | 3.56(dd), 3.66(dd) | CH_2_, CH |
| 28 | glycine | 3.58(s) | CH_2_ |
| 29 | N-phosphocreatine | 3.98(s) | CH_2_ |
| 30 | threonate | 4.03(dd) | 2-CH |
| 31 | uridine | 4.37(m), 5.94(t),7.88(d) | 2^׳^-CH, 5-CH, 6-CH |
| 32 | inosine | 4.30(m), 4.46(d), 4.78(t),  6.12(d), 8.26(s), 8.39(s) | 5-CH, 4-CH, 3-CH,  2-CH, 7-CH, 12-CH |
| 33 | glucose | 4.68(d), 5.28(d), 3.90(dd),  3.84(ddd), 3.48(m), 3.42(m), 3.29(m) | 1(*β*)-CH, 1(*α*)-CH,  6-CH_2_,3-CH,  4-CH, 2-CH |
| 34 | serine | 3.96(m) | 2-CH_2_ |
| 35 | guanosine | 4.76(d) | 3-CH |
| 36 | mannose | 5.22(d) | 1-CH |
| 37 | glycogen | 5.38(br, s) | 1-CH |
| 38 | glucose-1-phosphate | 5.45(m) | 1-CH |
| 39 | uracil | 5.82(d), 7.62(d) | 5-CH, 6-CH |
| 40 | ADP+ATP | 6.12(d), 8.26(s), 8.52(s) | 2-CH, 12-CH, 7-CH |
| 41 | fumarate | 6.53(s) | CH |
| 42 | anserine | 6.83(s) | 5-CH |
| 43 | tyramine | 6.94(d), 7.21(d) | 2-CH, 6-CH, 3-CH, 5-CH |
| 44 | phenylalanine | 7.35(d), 7.39(dd), 7.44(m) | 2-CH, 6-CH. 4-CH |
| 45 | thymine | 7.35(s), 1.93(s) | 5-CH_3_, 6-CH |
| 46 | xanthosine | 7.89(s) | 7-CH |
| 47 | xanthine | 7.95(s) | 2-CH |
| 48 | oxypurinol | 8.21(s) | 9-CH |
| 49 | hypoxanthine | 8.21(s), 8.26(s) | 2-CH, 7-CH |
| 50 | adenosine | 8.26(s), 8.39(s), 6.12(d) | 2-CH, 8-CH, 2^׳^-CH |
| 51 | formate | 8.48(s) | CHO |
| 52 | niacinamide | 8.97(s), 7.63(dd),  8.74(d), 8.28(m) | 2-CH, 5-CH,  6-CH, 4-CH |

Table S3 The differential metabolites based on ^1^H-NMR metabolomics analysis of liver tissue.

| Metabolites | ppm | M *vs* C | | | HAO *vs* M | | |
| --- | --- | --- | --- | --- | --- | --- | --- |
|  |  | P value | FC | Up/Down | P value | FC | Up/Down |
| 3-Hydroxybutyrate (4) | 4.15 | 0.003 | 1.73 | down | 0.015 | 1.38 | up |
| Lactate (5) | 1.34 | 0.003 | 1.47 | up | 0.014 | 0.63 | down |
| Acetate (8) | 1.94 | 0.027 | 1.49 | up | 0.048 | 0.71 | down |
| Succinate (12) | 2.41 | 0.000 | 1.88 | up | 0.0487 | 0.73 | down |
| Glutamine (16) | 3.76 | 0.001 | 0.64 | down | 0.001 | 1.26 | up |
| Creatine (18) | 3.95 | 0.009 | 0.56 | down | 0.005 | 1.56 | up |
| Choline (20) | 3.22 | 0.001 | 1.84 | up | 0.010 | 0.70 | down |
| Phosphorylcholine (21) | 3.24 | 0.000 | 1.68 | up | 0.501 | 0.70 | down |
| Carnitine (22) | 3.25 | 0.002 | 1.62 | up | 0.009 | 0.71 | down |
| TMAO (23) | 3.26 | 0.007 | 2.01 | up | 0.033 | 0.69 | down |
| GPC (24) | 3.28 | 0.000 | 0.71 | down | 0.000 | 1.29 | up |
| Taurine (25) | 3.29 | 0.044 | 0.61 | down | 0.010 | 2.05 | up |
| Glycerol (27) | 3.67 | 0.010 | 1.44 | up | 0.041 | 0.77 | down |
| Glycine (28) | 3.58 | 0.032 | 1.60 | up | 0.000 | 0.75 | down |
| Serine (34) | 3.97 | 0.005 | 0.58 | down | 0.043 | 1.49 | up |
| N-phosphocreatine (29) | 3.98 | 0.009 | 0.57 | down | 0.002 | 1.33 | up |
| Inosine (32) | 4.78 | 0.047 | 0.68 | down | 0.000 | 1.38 | up |
| Glucose (33) | 5.28 | 0.045 | 0.53 | down | 0.003 | 1.42 | up |
| Phenylalanine (44) | 7.35 | 0.006 | 0.78 | down | 0.000 | 1.9 | up |
| Hypoxanthine (49) | 8.26 | 0.047 | 0.79 | down | 0.611 | 1.83 | up |

The number of metabolites from NMR is identical to Table S2; FC: Fold Change.

Table S4 The differential lipids result of lipidomics in liver tissue based on UHPLC-Q/Orbitrap MS

| **No** | **Average Rt(min)** | **Average m/z** | **identification** | **Adduct** | **Formula** | **FC**  **(M vs C)** | **FC**  **(H vs M)** | **p value** | **Ontology** |
| --- | --- | --- | --- | --- | --- | --- | --- | --- | --- |
| **1** | 0.77 | 514.2843 | BA 24:1;O4;T | [M-H]^-^ | C_26_H_45_NO_7_S | 0.38 | 1.87 | 3.70E-02 | BileAcid |
| **2** | 1.59 | 342.3366 | NAE 19:0 | [M+H]^+^ | C_21_H_43_NO_2_ | 2.17 | 0.51 | 7.08E-05 | NAE |
| **3** | 2.18 | 370.3684 | NAE 21:0 | [M+H]^+^ | C_23_H_47_NO_2_ | 2.01 | 0.42 | 7.11E-05 | NAE |
| **4** | 2.20 | 558.3521 | LPC 19:1 | [M+Na]^+^ | C_27_H_54_NO_7_P | 2.03 | 0.28 | 7.41E-03 | LPC |
| **5** | 3.14 | 482.3230 | LPE 18:0 | [M+H]^+^ | C_23_H_48_NO_7_P | 2.19 | 0.64 | 1.14E-03 | LPE |
| **6** | 3.80 | 494.3604 | LPE O-20:1 | [M+H]^+^ | C_25_H_52_NO_6_P | 2.08 | 0.54 | 3.11E-03 | EtherLPE |
| **7** | 4.14 | 656.4736 | LPC 28:4 | [M+H]^+^ | C_36_H_66_NO_7_P | 0.46 | 1.70 | 3.94E-02 | LPC |
| **8** | 4.34 | 321.2795 | FA 21:2 | [M-H]^-^ | C_21_H_38_O_2_ | 0.49 | 2.42 | 8.92E-03 | FA |
| **9** | 4.49 | 791.4628 | PA 22:6_22:6 | [M-H]^-^ | C_47_H_69_O_8_P | 2.09 | 0.73 | 8.15E-06 | PA |
| **10** | 5.26 | 616.4603 | SL 34:1;0O | [M-H]^-^ | C_34_H_67_NO_6_S | 2.01 | 0.69 | 2.40E-04 | SL |
| **11** | 5.37 | 819.5214 | PG 18:1_22:6 | [M-H]^-^ | C_46_H_77_O_10_P | 2.19 | 0.71 | 5.69E-08 | PG |
| **12** | 5.41 | 618.4779 | SL 34:0;0O | [M-H]^-^ | C_34_H_69_NO_6_S | 2.24 | 0.71 | 3.31E-08 | SL |
| **13** | 5.61 | 795.5175 | PG 18:1_20:4 | [M-H]^-^ | C_44_H_77_O_10_P | 2.03 | 0.81 | 1.34E-07 | PG |
| **14** | 5.77 | 604.4609 | SL 33:0;0O | [M-H]^-^ | C_33_H_67_NO_6_S | 2.10 | 0.68 | 3.33E-07 | SL |
| **15** | 5.88 | 654.5107 | DG 18:3_20:5 | [M+NH_4_]^+^ | C_41_H_64_O_5_ | 0.49 | 1.44 | 6.51E-04 | DG |
| **16** | 5.98 | 632.4949 | SL 35:0;0O | [M-H]^-^ | C_35_H_71_NO_6_S | 2.32 | 0.59 | 7.35E-07 | SL |
| **17** | 6.91 | 535.4321 | DG 28:0 | [M+Na]^+^ | C_31_H_60_O_5_ | 0.50 | 2.73 | 4.69E-02 | DG |
| **18** | 7.10 | 873.5388 | PI 18:0_19:3 | [M-H]^-^ | C_46_H_83_O_13_P | 3.16 | 0.70 | 2.12E-07 | PI |
| **No** | **Average Rt(min)** | **Average m/z** | **identification** | **Adduct** | **Formula** | **FC**  **(M vs C)** | **FC**  **(H vs M)** | **p value** | **Ontology** |
| **19** | 7.48 | 784.5527 | PE 18:0_20:3;O | [M-H]^-^ | C_43_H_80_NO_9_P | 0.46 | 1.27 | 3.44E-13 | OxPE |
| **20** | 7.66 | 523.4516 | VAE 16:1 | [M+H]^+^ | C_36_H_58_O_2_ | 0.30 | 2.05 | 2.11E-04 | VAE |
| **21** | 7.76 | 676.6697 | Cer 25:0;2O/19:1;O | [M+H-H_2_O]^+^ | C_44_H_87_NO_4_ | 0.37 | 2.71 | 6.08E-03 | Cer_HDS |
| **22** | 7.99 | 423.3121 | DG 20:0 | [M+Na]^+^ | C_23_H_44_O_5_ | 8.98 | 0.44 | 4.78E-10 | DG |
| **23** | 8.00 | 283.2647 | FA 18:0 | [M-H]^-^ | C_18_H_36_O_2_ | 2.03 | 0.66 | 2.51E-04 | FA |
| **24** | 9.06 | 952.7622 | TG 16:0_22:6_18:2;2O | [M+NH_4_]^+^ | C_59_H_98_O_8_ | 0.38 | 2.08 | 4.94E-04 | OxTG |
| **25** | 9.34 | 960.7635 | TG 18:2_22:6_18:2;1O | [M+NH_4_]^+^ | C_61_H_98_O_7_ | 0.30 | 1.78 | 1.27E-05 | OxTG |
| **26** | 9.42 | 760.5828 | PC 34:1 | [M+H]^+^ | C_42_H_82_NO_8_P | 3.29 | 0.50 | 5.13E-04 | PC |
| **27** | 9.42 | 734.5698 | PE 35:0 | [M+H]^+^ | C_40_H_80_NO_8_P | 2.19 | 0.37 | 1.16E-02 | PE |
| **28** | 9.44 | 904.7616 | TG 16:0_18:1_18:3;2O | [M+NH_4_]^+^ | C_55_H_98_O_8_ | 0.45 | 1.64 | 1.01E-04 | OxTG |
| **29** | 9.47 | 988.7371 | TG 20:5_20:5_22:6 | [M+NH_4_]^+^ | C_65_H_94_O_6_ | 0.32 | 1.84 | 8.72E-05 | TG |
| **30** | 9.56 | 853.7244 | SM 45:3;2O | [M+H]^+^ | C_50_H_97_N_2_O_6_P | 0.36 | 1.50 | 4.57E-07 | SM |
| **31** | 9.61 | 886.7466 | TG 16:0_18:2_18:3;1O | [M+NH_4_]^+^ | C_55_H_96_O_7_ | 0.46 | 1.36 | 1.16E-06 | OxTG |
| **32** | 9.73 | 919.6753 | TG 18:2_16:3_22:6 | [M+Na]^+^ | C_59_H_92_O_6_ | 0.34 | 1.90 | 1.53E-07 | TG |
| **33** | 9.87 | 945.6892 | TG 18:2_20:5_20:5 | [M+Na]^+^ | C_61_H_94_O_6_ | 0.36 | 1.50 | 1.75E-07 | TG |
| **34** | 9.91 | 945.6967 | TG 18:2_18:4_22:6 | [M+Na]^+^ | C_61_H_94_O_6_ | 0.37 | 1.51 | 3.89E-07 | TG |
| **35** | 10.76 | 801.6951 | TG 14:0_16:0_16:0 | [M+Na]^+^ | C_49_H_94_O_6_ | 2.19 | 0.48 | 1.72E-03 | TG |
| **36** | 18.08 | 820.7381 | TG 14:0_16:0_18:2 | [M+NH_4_]^+^ | C_51_H_94_O_6_ | 2.66 | 0.53 | 9.72E-03 | TG |
| **37** | 18.11 | 795.6256 | SM 41:4;2O | [M+H]^+^ | C_46_H_87_N_2_O_6_P | 2.00 | 0.52 | 1.60E-02 | SM |
| **No** | **Average Rt(min)** | **Average m/z** | **identification** | **Adduct** | **Formula** | **FC**  **(M vs C)** | **FC**  **(H vs M)** | **p value** | **Ontology** |
| **38** | 18.14 | 813.6830 | SM 18:1;2O/24:1 | [M+H]^+^ | C_47_H_93_N_2_O_6_P | 2.11 | 0.57 | 2.19E-02 | SM |
| **39** | 18.14 | 794.7212 | TG 14:0_16:0_16:1 | [M+NH_4_]^+^ | C_49_H_92_O_6_ | 2.59 | 0.37 | 3.21E-02 | TG |
| **40** | 18.68 | 801.6852 | SM 18:1;2O/23:0 | [M+H]^+^ | C_46_H_93_N_2_O_6_P | 2.02 | 0.35 | 1.35E-02 | SM |
| **41** | 18.69 | 848.7687 | TG 16:0_16:1_18:1 | [M+NH_4_]^+^ | C_53_H_98_O_6_ | 4.42 | 0.63 | 2.69E-05 | TG |
| **42** | 18.70 | 520.5086 | Cer 18:1;2O/16:0 | [M+H-H_2_O]^+^ | C_34_H_67_NO_3_ | 2.44 | 0.53 | 4.26E-03 | Cer_NS |
| **43** | 18.70 | 630.6178 | Cer 18:1;2O/24:1 | [M+H-H_2_O]^+^ | C_42_H_81_NO_3_ | 3.76 | 0.51 | 6.16E-04 | Cer_NS |
| **44** | 18.70 | 930.8234 | TG 18:1_18:1_19:2;1O | [M+NH_4_]^+^ | C_58_H_104_O_7_ | 4.15 | 0.65 | 2.67E-05 | OxTG |
| **45** | 18.71 | 768.5531 | PE 18:0_20:4 | [M+H]^+^ | C_43_H_78_NO_8_P | 2.33 | 0.58 | 6.34E-03 | PE |
| **46** | 18.75 | 636.6280 | Cer 18:1;2O/23:0 | [M+H]^+^ | C_41_H_81_NO_3_ | 4.08 | 0.36 | 2.86E-03 | Cer_NS |
| **47** | 18.76 | 622.6144 | Cer 18:1;2O/22:0 | [M+H]^+^ | C_40_H_79_NO_3_ | 4.36 | 0.42 | 3.05E-03 | Cer_NS |
| **48** | 18.80 | 634.6141 | Cer 18:1;2O/23:0;O | [M+H-H_2_O]^+^ | C_41_H_81_NO_4_ | 4.35 | 0.32 | 4.38E-03 | Cer_HS |
| **49** | 18.83 | 716.5198 | PE 34:2 | [M+H]^+^ | C_39_H_74_NO_8_P | 2.07 | 0.54 | 2.15E-02 | PE |
| **50** | 18.83 | 792.5524 | PE 40:6 | [M+H]^+^ | C_45_H_78_NO_8_P | 2.04 | 0.59 | 2.03E-02 | PE |
| **51** | 18.84 | 768.5506 | PE 38:4 | [M+H]^+^ | C_43_H_78_NO_8_P | 2.41 | 0.54 | 6.98E-03 | PE |
| **52** | 19.07 | 853.7234 | TG 16:0_16:0_18:2 | [M+Na]^+^ | C_53_H_98_O_6_ | 2.94 | 0.69 | 4.05E-05 | TG |
| **53** | 19.09 | 976.8325 | TG 20:1_18:2_22:5 | [M+NH_4_]^+^ | C_63_H_106_O_6_ | 2.86 | 0.62 | 5.99E-04 | TG |

According to the accurate molecular ion masses, the retention times, and the standard compounds in HMDB database.
